# Morphology and Surface Reconstruction-Driven Catalytic Enhancement in CoMn_2_O_4_ for Efficient OER Application

**DOI:** 10.3390/ma19030542

**Published:** 2026-01-29

**Authors:** Abu Talha Aqueel Ahmed, Abu Saad Ansari, Sangeun Cho, Atanu Jana

**Affiliations:** 1 Division of System Semiconductor, Dongguk University, Seoul 04620, Republic of Korea; 2 Nano Center Indonesia Research Institute, Puspiptek Street, South Tangerang 15314, Banten, Indonesia

**Keywords:** nanograss, OER, CoMn_2_O_4_, electrocatalyst, intrinsic activity, electrochemical kinetics, overall-water electrolysis

## Abstract

The development of efficient and durable oxygen evolution reaction (OER) catalysts from earth-abundant materials is essential for advancing alkaline water electrolysis. Herein, nanograss-like CoMn_2_O_4_ electrode films are directly grown on stainless-steel substrates via a temperature-controlled hydrothermal approach, and their OER performance is systematically investigated. The CoMn_2_O_4_ obtained at 120 °C (CMO-120) delivers the best catalytic activity in 1.0 M KOH, requiring an overpotential of 292 mV at 10 mA cm^−2^, which is lower than those synthesized at 150 (CMO-150) and 90 °C (CMO-90). Notably, activity of CMO-120 becomes even more pronounced at elevated current densities, achieving the low overpotential of 434 mV even at 300 mA cm^−2^, substantially outperforming both CMO-90 and CMO-150 electrodes. The enhanced activity is attributed to an interconnected nanograss architecture with mixed Co^2+^/Co^3+^ and Mn^2+^/Mn^3+^ redox couples and abundant defect-related oxygen species, which result in increased electrochemically active surface area and improved charge transportation throughout the nanograss architecture that facilitate OH^−^ adsorption and OER intermediate transformation. Furthermore, CMO-120 demonstrates excellent durability (100 h) after electro-oxidation-induced surface activation. These findings highlight precise temperature regulation as an effective strategy for optimizing Mn-Co spinel for efficient alkaline OER applications.

## 1. Introduction

The global transition toward a sustainable energy infrastructure has intensified the demand for efficient, cost-effective, and durable technologies for renewable hydrogen production [1]. Among electrochemical water-splitting processes, the oxygen evolution reaction (OER) remains a major performance bottleneck due to its intrinsically sluggish four-electron transfer mechanism and the high overpotentials required to drive the reaction [2,3]. Consequently, the development of electrocatalysts that can accelerate OER kinetics while maintaining structural and electrochemical stability under high-current operation is essential for advancing electrolyzer efficiency in both industrial and renewable energy systems [4]. Although noble metal oxides such as IrO_2_ and RuO_2_ are widely regarded as benchmark OER catalysts because of their high intrinsic activity, their scarcity and high cost severely limit large-scale practical deployment [5,6]. This challenge has driven extensive research into earth-abundant transition-metal-based materials, including oxides, hydroxides, sulfides, and spinel structures, which offer competitive catalytic performance with improved economic viability [7].

Within this context, spinel oxides (AB_2_O_4_), where A and B are transition-metal cations, have attracted significant attention as promising OER electrocatalysts owing to their tunable electronic structures, compositional flexibility, and relatively high electrical conductivity among non-noble catalysts [8,9,10]. By modulating the type and distribution of metal cations at tetrahedral (A) and octahedral (B) sites, the surface redox behavior and charge-transport properties of spinel oxides can be systematically tailored to optimize active-site availability and reaction kinetics [11]. For example, Grimaud et al. demonstrated that tuning B-site occupancy in Co-based spinel modulates metal–oxygen covalency, which directly correlates with enhanced OER activity [12]. Similarly, Suntivich et al. established that the intrinsic OER activity of AB_2_O_4_ spinel is governed by the electronic configuration of octahedrally coordinated B-site cations, providing a fundamental descriptor for rational catalyst design [13]. Furthermore, Ya et al. showed that temperature-regulated surface engineering of MgCo_2_O_2_ spinel effectively alters surface valence states and composition, leading to a marked reduction in OER overpotential [14]. Complementary theoretical and in situ studies have also revealed that structural parameters such as oxygen-vacancy concentration, cation distribution, and metal–oxygen bond covalency play critical roles in governing OER activity in spinel systems [15].

Among earth-abundant transition-metal-based electrocatalysts, Mn- and Co-based materials have been extensively investigated for OER owing to their rich redox chemistry, favorable stability in alkaline media, and low cost [16]. Cobalt-based oxides and (oxy)hydroxides exhibit high intrinsic OER activity due to reversible Co^2+^/Co^3+^ redox transitions and strong metal–oxygen covalency that facilitates adsorption of oxygenated intermediates [17]. Manganese-based catalysts, inspired by the Mn_4_Ca cluster in natural photosystem II, offer excellent structural robustness and multivalent redox behavior (Mn^2+^/Mn^3+^), contributing to long-term durability under anodic polarization [12,18]. The integration of Co and Mn into bimetallic systems enables synergistic electronic interactions and charge redistribution between the two metal centers, optimizing adsorption energies of OER intermediates and thereby enhancing catalytic kinetics beyond those of single-metal counterparts [19,20,21,22,23,24]. In addition to composition, catalyst morphology plays a decisive role in determining OER performance. Morphological engineering has emerged as an effective strategy to increase electrochemically active surface area, shorten ion-diffusion pathways, and improve electron transport [25]. In particular, one-dimensional (1D) nanostructures such as nanowires and nanorods offer distinct advantages, including continuous electron-conduction pathways, high aspect ratios, and abundant exposed active sites, which collectively promote faster charge transfer and improved catalytic efficiency compared with bulk, nanoparticle, or dense film morphologies [26,27,28].

Despite these advances and the growing literature on CoMn_2_O_4_ spinel electrocatalysts for alkaline OER, existing studies have largely focused on compositional tuning or post-treatment strategies, while the direct and systematic correlation between hydrothermal growth temperature and high-current OER performance remains insufficiently explored [29,30,31]. In particular, how temperature-regulated nucleation and anisotropic growth govern the formation of vertically aligned nanograss architectures and their impact on charge-transfer kinetics and long-term stability is still not well understood. In this work, we address this gap by directly growing CoMn_2_O_4_ electrode films on stainless-steel substrates via a temperature-controlled hydrothermal route without templates or binders, enabling precise modulation of nanograss morphology through reaction temperature alone. By comparatively investigating samples synthesized at different temperatures, we demonstrate that optimized nanograss-like architectures significantly enhance OER activity not only at conventional current densities (10 mA cm^−2^) but also under practically relevant high-current operation (up to 300 mA cm^−2^). Furthermore, the study elucidates the role of temperature-induced phase evolution toward surface oxyhydroxide species during initial activation and its contribution to sustained catalytic durability. These findings establish temperature-guided morphology engineering as an effective and scalable strategy for unlocking the full OER potential of Mn-Co spinel oxides, providing new insight into the rational design of robust, high-rate oxygen evolution electrodes.

## 2. Materials and Methods

### 2.1. Materials

Urea (CO(NH_2_)_2_), potassium hydroxide (KOH, ≥85%), manganese(II) nitrate hydrate (Mn(NO_3_)_2_·xH_2_O), ethanol (CH_3_CH_2_OH, ≥95%), and cobalt(II) nitrate hexahydrate (Co(NO_3_)_2_·6H_2_O) were purchased from Sigma-Aldrich (St. Louis, MO, USA) and used as received without any further purification. Deionized water prepared in the laboratory was used for all solution preparations and in washing processes. Commercial stainless-steel substrates were employed as the conductive supports for the hydrothermal growth of the electrode films.

### 2.2. Synthesis of CoMn_2_O_4_ Electrodes

**Figure 1:**
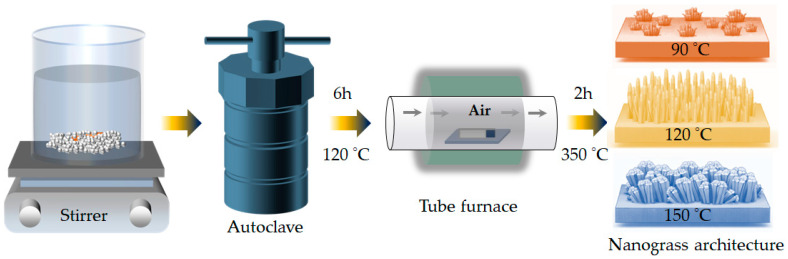
Schematic illustration of the hydrothermal synthesis of nanograss-like CoMn_2_O_4_ electrode films on stainless-steel substrates. In the first step (I), Co^2+^ and Mn^2+^ precursor species undergo hydrolysis and homogeneous nucleation in an aqueous solution containing urea under hydrothermal conditions. In the subsequent step (II), the as-grown precursor structures undergo annealing to form the spinel CoMn_2_O_4_ phase with stable adhesion and uniform coverage. By adjusting the hydrothermal temperature, the nucleation density and growth kinetics are regulated, enabling precise control over nanograss morphology and surface architecture.

The CoMn_2_O_4_ (CMO) electrode films were synthesized via a hydrothermal deposition process directly on stainless-steel substrates. Prior to hydrothermal synthesis, the stainless-steel substrates were cut into appropriate dimensions (1 × 5 cm^2^) and mechanically roughened using successive grades of polishing paper to increase surface roughness and promote strong adhesion of the deposited catalyst layer. The roughened substrates were then thoroughly rinsed with deionized water to remove residual debris generated during polishing, followed by repeated rinsing with ethanol and deionized water. The cleaned substrates were finally dried in air and directly used for the hydrothermal growth of the CMO films (Figure 1). In a typical procedure, stoichiometric amounts of Co(NO_3_)_3_·6H_2_O and Mn(NO_3_)_2_·xH_2_O were dissolved in 50 mL of deionized water to maintain a Co:Mn molar ratio of 1:2, corresponding to the targeted CoMn_2_O_4_ spinel composition. Subsequently, 15 mmol of CO(NH_2_)_2_ was added as a homogeneous precipitation agent and the mixed solution was stirred until a clear and uniform precursor solution was obtained. The precursor solution was transferred into a Teflon-lined stainless-steel autoclave and the pretreated stainless-steel substrates were placed vertically in the solution with the polished surface facing inward. The autoclave was sealed and maintained at 120 °C for 6 h to obtain the CoMn_2_O_4_ electrode. After completion of the hydrothermal reaction, the autoclave was allowed to cool naturally to room temperature. The substrates were removed and thoroughly rinsed with deionized water to eliminate residual reactants and dried at 80 °C overnight in a vacuum oven. The as-deposited precursor films were subsequently annealed in an air atmosphere at 350 °C for 2 h with a heating rate of 2 °C min^−1^ to obtain the crystalline CoMn_2_O_4_ spinel structure and denoted as CMO-120. After annealing, the electrodes were allowed to cool naturally to room temperature and were directly used for further characterization and electrochemical evaluation. During the hydrothermal process, the gradual thermal decomposition of urea provides a controlled supply of hydroxide (OH^−^) ions:

CO(NH_2_)_2_ + H_2_O → 2NH_3_ + CO_2_, (1)

CO_2_ + 2(OH)^−^ → (CO_3_)^2−^ + H_2_O, (2)

NH_3_ + H_2_O ⇌ (NH_4_)^+^ + (OH)^−^ (3)

The gradual release of OH^−^ ensures homogeneous nucleation and uniform growth of precursor species on the stainless-steel substrate:

Co(NO_3_)_2_ → Co^2+^ + 2(NO_3_)^−^, (4)

Mn(NO_3_)_2_ → Mn^2+^ + 2(NO_3_)^−^, (5)

Co^2+^ + (CO_3_)^2−^ + 2(OH)^−^ → Co(OH)_2_(CO_3_), (6)

Mn^2+^ + (CO_3_)^2−^ + 2(OH)^−^ → Mn(OH)_2_(CO_3_) (7)

This mixed hydroxide precursor uniformly covers the substrate surface due to heterogeneous nucleation on the stainless-steel surface and upon annealing the hydroxide precursor undergoes dehydration and oxidation, leading to spinel CoMn_2_O_4_ formation, as follows:

Co(OH)_2_(CO_3_) + 2Mn(OH)_2_(CO_3_) → CoMn_2_O_4_ + 3CO_2_ + O_2_ + 3H_2_O (8)

To investigate the influence of synthesis temperature on crystal growth, morphology evolution, and electrocatalytic properties, the hydrothermal deposition was further carried out at elevated temperatures of 150 °C and 90 °C under the identical experimental conditions, and the obtained films were denoted as CMO-150 and CMO-90, respectively. Increasing the hydrothermal temperature is expected to accelerate nucleation and growth kinetics, modify particle coalescence and crystallinity, and thereby influence the structural and electrochemical characteristics of the resulting CoMn_2_O_4_ electrodes.

### 2.3. Material Characterization

The crystalline phase and structural identification of the synthesized CoMn_2_O_4_ electrode were examined using the X-ray diffraction (XRD) technique with the help of Rigaku Smartlab instrument (Rigaku, Tokyo, Japan) (operational voltage of 40 kV and current of 30 mA). The XRD spectrum was recorded with a Cu Kα radiation source (λ = 1.5406 Å) over an 2θ spectral range of 15–70° at a scan rate of 2° min^−1^. The surface morphology and microstructural features of the electrodes were analyzed by field-emission scanning electron microscopy (FESEM) using the JEOL JSM-6701F instrument (JEOL, Tokyo, Japan). Elemental composition was further verified using energy-dispersive X-ray spectroscopy (EDX) detector (Bruker AXS Microanalysis GmbH, Berlin, Germany) attached to the FESEM system. The surface chemical states and electronic structure of the electrodes were investigated by X-ray photoelectron spectroscopy (XPS) using a ULVAC PHI 5000 VersaProbe instrument (ULVAC-PHI, Inc., Chigasaki, Japan). High-resolution spectra of Co 2p, Mn 2p, and O 1s regions were recorded to determine oxidation states, metal–oxygen bonding environments, and the presence of defect-related oxygen species. All binding energies were calibrated using the contaminant carbon (C 1s) peak at 284.51 eV as a reference.

### 2.4. Catalytic OER Activity

The OER performance of the CoMn_2_O_4_ electrodes were evaluated using a standard three-electrode electrochemical configuration connected to an electrochemical workstation (VersaSTAT, Ametek Scientific Instruments, Berwyn, PA, USA). The formed CoMn_2_O_4_ electrode films grown on stainless-steel substrates were used as the working electrodes. A platinum mesh and a saturated calomel electrode (SCE, occupied with the saturated KCl solution) were employed as the counter and reference electrodes, respectively. All electrochemical measurements were conducted in 1.0 M KOH aqueous electrolyte at room temperature. The linear sweep voltammetry (LSV) curves were carried out at a scan rate of 1.0 mV s^−1^ in the potential window of 0.0 to 1.3 V (vs. SCE). The measured potentials were converted to the reversible hydrogen electrode (RHE) scale according to the following equation [32]:

*E*_RHE_ = *E*_SCE_° + *E*_SCE_ + (*pH* × 0.059), (9)

where *E*_RHE_ is the converted potential in RHE scale. *E*_SCE_ and *E*_SCE_° are the obtained potential in SCE scale and the standard potential of reference electrode at an ambient room temperature. To eliminate ohmic losses, all the obtained polarization curves were corrected for solution resistance using *iR*-compensation (*J*·*R*_S_ correction), where *R*_S_ was obtained from electrochemical impedance spectroscopy (EIS) analysis. The OER overpotential (*η*) was determined from the *iR*-compensated polarization curves by subtracting the thermodynamic OER potential (1.23 V vs. RHE) from the applied potential required to reach a given current density using the following equations [22]:

*E*_RHE_ (*J*·*R*_S_ correction) = *E*_RHE_ − (*J*·*R*_S_), (10)

*η* = *E*_RHE_ (*J*·*R*_S_ correction) − 1.23, (11)

Thereafter, the Tafel slopes were derived from the linear region of the corresponding Tafel plots (*η* vs. log *J*) to analyze the reaction kinetics, which is expressed by the following equation [33]:

*η* = *a* + (*b* × log(*J*)), (12)

where *a* and *b* are the arbitrary constant and Tafel slope of the curve, respectively. The electrochemical impedance spectroscopy measurements were carried out at the OER operating potential over a frequency range of 10 mHz to 10 kHz with an AC amplitude of 10 mV to evaluate charge-transfer resistance and interfacial kinetics. The impedance data were fitted using an equivalent circuit model to assess the solution resistance and charge transfer resistance (*R*_ct_). Further, the electrochemically active surface area (*ECSA*) was estimated by measuring the double-layer capacitance (*C*_dl_) from cyclic voltammetry (CV) recorded in a non-faradaic potential region at various scan rates (10–50 mV s^−1^). The *ECSA* was then calculated using the following equation:

*ECSA* = *C*_dl_/*C*_e_, (13)

where *C*_e_ is the specific capacitance of a smooth surface in alkaline electrolyte. The long-term electrochemical stability of the optimized electrode was assessed by chronopotentiometry test at a constant applied current density 100 mA cm^−2^ for an extended duration of 100 h. All measurements were repeated to ensure the reproducibility of the OER activity.

## 3. Results

### 3.1. Morphological and Compositional Properties of CoMn_2_O_4_ Electrode Films

**Figure 2:**
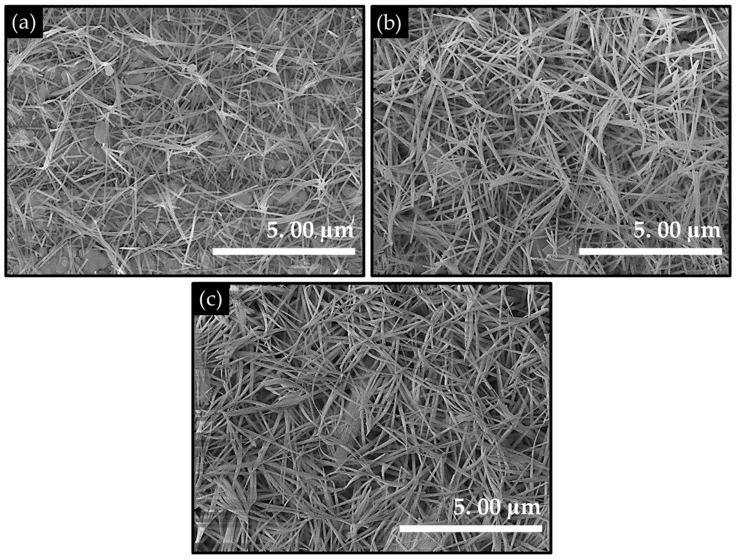
FESEM images of the CoMn_2_O_4_ electrode films for various deposition temperatures associated with the (**a**) CMO-90, (**b**) CMO-120, and (**c**) CMO-150 samples.

The FESEM imaging was employed to examine the effect of hydrothermal synthesis temperature on nanoscale growth behavior, surface texture, and film coverage on the stainless-steel substrate. As shown in Figure 2, all samples exhibit a distinct one-dimensional nanograss-like architecture uniformly grown on the stainless-steel substrate, indicating effective heterogeneous nucleation and strong substrate adhesion induced by surface roughening. The FESEM image of CMO-90 film (Figure 2a) shows that the electrode surface is covered with relatively thin and sparsely packed nanograss structures, reflecting the early stage of nucleation and limited growth kinetics at lower hydrothermal temperature. The nanograss architectures are non-uniform in the distribution with noticeable gaps between the adjacent structures. This incomplete coverage suggests insufficient precursor decomposition and slower ion diffusion, which restrict the vertical growth and lateral interconnection of nanostructures. Upon increasing the synthesis temperature (CMO-120, Figure 2b), a well-defined, dense, and highly interconnected nanograss network is formed. The nanograss structures are uniformly distributed across the substrate, exhibiting high aspect ratios with typical lengths in the range of ~1.3–3.9 µm and an average diameter of ~40–70 nm. The nanograss intersect and overlap to each other creating an open yet mechanically robust framework. This morphology maximizes surface exposure, generates abundant edge and tip sites, and forms continuous ion-transport channels throughout the electrode. The homogeneous growth and optimal density of nanograss in CMO-120 film indicate a balanced nucleation-growth process, where enhanced thermal energy promotes controlled anisotropic growth without inducing excessive aggregation. In contrast, the CMO-150 film (Figure 2c) exhibits the signs of overgrowth and partial structural congestion. Although the nanograss morphology is preserved, the structures appear thicker and more densely stacked, with slightly reduced intergrass spacing. The excessive growth at higher temperature likely accelerates precursor decomposition and crystal growth rates, leading to coalescence and partial loss of accessible surface area. This densification can impede the electrolyte penetration and gas diffusion, which may adversely affect electrochemical performance. The formation of the nanograss architecture can be attributed to urea-assisted slow OH^−^ and CO_3_^2−^ release, enabling gradual co-precipitation of the Co-Mn hydroxy-carbonate species on the stainless-steel surface. The roughened substrate provides abundant nucleation sites, while temperature-controlled hydrothermal conditions govern the anisotropic growth of one-dimensional nanostructures. At 120 °C, the growth kinetics are optimized to favor the vertical elongation and uniform distribution, whereas excessive thermal energy at 150 °C induces rapid growth and structural densification.

The EDX analysis was performed to verify the elemental composition and confirm the successful incorporation of Co and Mn within the CoMn_2_O_4_ nanograss electrodes grown on stainless-steel substrates. For all the electrode films, the EDX spectra clearly show the presence of Co, Mn, and O, confirming the formation of cobalt–manganese oxide-based films. The Co and Mn signals observed for CMO-90 film (Figure S1a) confirm that the nanograss layer formed even at the lowest hydrothermal temperature. However, the relatively weaker Co/Mn intensities are attributed to the lower surface coverage and thinner deposited layer, which are in agreement with the FESEM results. With increasing the synthesis temperature to 120 °C (CMO-120, Figure S1b), the EDX spectrum exhibits more pronounced Co and Mn signals, which are consistent with the dense and interconnected morphology. Importantly, the Co:Mn atomic ratio estimated from the EDX analysis remains close to the targeted 1:2 stoichiometric ratio, supporting the intended CoMn_2_O_4_ composition. For CMO-150 film (Figure S1c), the Co and Mn peaks remain evident, confirming retention of the Co-Mn oxide phase at higher synthesis temperature. The comparatively stronger overall oxide-related signal is consistent with increased growth and densification of the nanograss layer at elevated temperature. Overall, the EDX spectral analysis confirms the successful deposition of Co-Mn-O electrodes on stainless steel and support that the compositional integrity is maintained across the temperature series, enabling reliable comparison of morphology–performance relationships.

### 3.2. Crystallographic and Bonding Properties of CoMn_2_O_4_ Electrode Film

**Figure 3:**
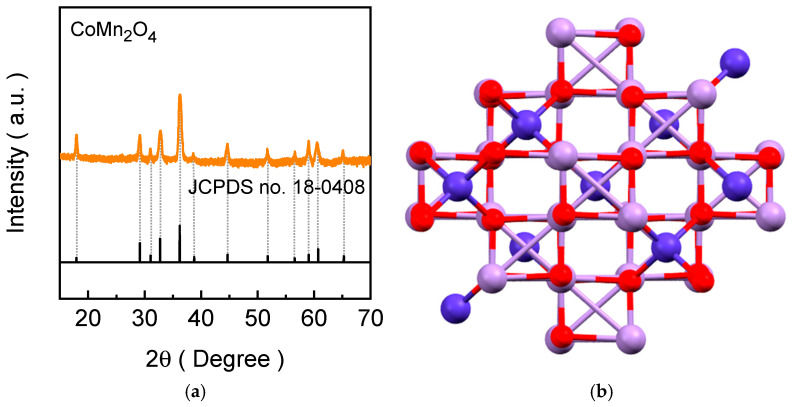
(**a**) XRD spectra along with the relevant JCPDS reference pattern (card number #18-0408) and (**b**) crystallographic schematic arrangement for the tetragonal CoMn_2_O_4_ electrode films. Notably, in the crystal structure model, Co, Mn, and O atoms are shown in blue, light violet, and red colors.

The crystal structure and phase purity of the synthesized CoMn_2_O_4_ electrode was investigated by the XRD spectral analysis (Figure 3). The XRD pattern of the CMO-120 film (Figure 3a) exhibits a series of characteristic diffraction peaks, matching well with the reference JCPDS card No. 18-0408, which can be indexed to the tetragonal spinel CoMn_2_O_4_ phase. The distinct diffraction peaks located at approximately 2θ ≈ 18.9°, 31.2°, 36.8°, 44.7°, 59.2°, and 65.1° correspond to the (111), (220), (311), (400), (511), and (440) crystallographic planes of spinel CoMn_2_O_4_, respectively. These reflections are in good agreement with the characteristic crystallographic planes of tetragonally distorted CoMn_2_O_4_, confirming the successful phase formation. Importantly, no additional peaks related to secondary cobalt or manganese oxide phases are detected, indicating the high phase purity of the synthesized electrode. Figure 3b illustrates the crystal structure of tetragonal CoMn_2_O_4_, highlighting the cation arrangement within the distorted spinel lattice. In this structure, Co^2+^ ions primarily occupy the tetrahedral sites, while Mn^3+^ ions are located at the octahedral sites, coordinated by oxygen atoms. The tetragonal distortion, compared to an ideal cubic spinel, originates from the Jahn-Teller activity of Mn^3+^, which elongates the MnO_6_ octahedra along the c-axis and lowers the symmetry. This structural distortion modifies the metal-oxygen bond lengths and electronic environment, which is known to influence redox behavior and catalytic activity during the oxygen evolution reaction. The excellent agreement between the experimental XRD pattern and the tetragonal crystal model confirms that the hydrothermal synthesis followed by mild annealing yields a structurally well-defined tetragonal CoMn_2_O_4_ spinel. This crystallographic framework provides multiple redox-active Co and Mn centers and flexible metal–oxygen bonding, which are essential for facilitating oxygen evolution kinetics.

### 3.3. Chemical Bonding States of CoMn_2_O_4_ Electrode Film

**Figure 4:**
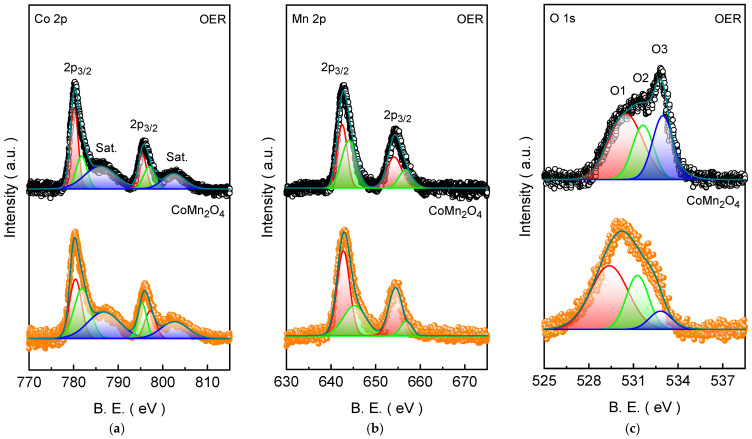
High-resolution XPS spectra of CoMn_2_O_4_ electrode film measured before and after the OER test for the (**a**) Co 2p, (**b**) Mn 2p, and (**c**) O 1s emissions. All high-resolution emission spectra were deconvoluted using Gaussian fitting functions.

To investigate the surface electronic structure and oxidation states of the CoMn_2_O_4_ electrode film, detailed XPS analysis was performed. The spectrum confirms that Co, Mn, and O are the primary elements present along with the additional C 1s feature aroused from the contaminant carbon present inside the chamber. The absence of other impurity peaks indicates the high surface purity of the CoMn_2_O_4_ film. The high-resolution Co 2p spectrum of the CMO-120 film (Figure 4a) exhibits the characteristic spin–orbit doublet of Co 2p_3/2_ and Co 2p_1/2_, together with two shake-up satellite features, confirming the presence of cobalt in a mixed oxidation environment typical of spinel oxides. The main Co 2p_3/2_ and Co 2p_1/2_ peaks are centered at 780.41 and 795.98 eV, respectively, giving a spin–orbit separation of ~15.6 eV, which is consistent with cobalt oxide lattices [29]. In addition, two pronounced satellite (Sat.) peaks located at 786.95 and 802.65 eV are clearly observed. The appearance of these satellites is a well-known fingerprint of Co^2+^ species due to final-state shake-up processes, indicating that Co^2+^ is present on the surface. To further resolve the cobalt chemical states, the Co 2p spectrum was deconvoluted into multiple components. The Co 2p_3/2_ region can be fitted with two peaks at 780.42 and 781.94 eV, which are assigned to Co^3+^ and Co^2+^, respectively. Similarly, the Co 2p_1/2_ region is deconvoluted into two corresponding components at 795.34 (Co^3+^) and 797.18 eV (Co^2+^). The coexistence of Co^3+^ and Co^2+^ strongly supports the formation of a spinel-type Co-Mn oxide, where cobalt typically occupies tetrahedral coordination environments. This mixed Co^3+^/Co^2+^ surface chemistry is highly advantageous for alkaline OER because cobalt centers can readily undergo oxidation to higher-valence states under anodic polarization (e.g., Co^2+^ → Co^3+^/CoOOH-like species), which are widely regarded as the catalytically active forms during oxygen evolution [34]. Meanwhile, the presence of Co^2+^ (supported by the strong satellite intensity) implies enhanced redox flexibility and charge compensation within the lattice, which can facilitate electron transfer and promote the formation/turnover of oxygenated intermediates.

The high-resolution Mn 2p XPS spectrum (Figure 4b) shows two well-resolved spin–orbit split components at approximately 642.98 (Mn 2p_3/2_) and 654.46 eV (Mn 2p_1/2_), with a characteristic spin–orbit separation of ~11.5 eV. These binding energies and the spectral shape are consistent with manganese oxide environments in mixed oxidation states [35]. The deconvolution of the Mn 2p_3/2_ and Mn 2p_1/2_ peak reveals two distinct doublets located at 642.82 and 654.41 eV (Mn^2+^) and 645.30 eV and 656.84 eV (Mn^3+^), respectively. The presence of both Mn^2+^ and Mn^3+^ indicates that manganese exists in a mixed valence state in the CoMn_2_O_4_ structures. This analysis aligns with earlier reports on manganese-containing spinel oxides, where the electronic configuration of Mn^2+^ (3d^5^) and Mn^3+^ (3d^4^) in the octahedral environment of the spinel facilitates redox flexibility and can strongly influence catalytic behavior [36]. Further, the Mn^2+^ sites are often associated with lower binding energies due to a higher electron density around the Mn center, whereas Mn^3+^ features arise at higher binding energies due to increased effective nuclear charge and partial oxidation [37]. The coexistence of Mn^2+^ and Mn^3+^ in CMO-120 film suggests a balanced redox environment where both oxidation states can participate in electron exchange during OER, potentially enhancing catalytic turnover [38]. Moreover, the presence of mixed Mn valence states can influence the degree of lattice distortion and facilitate oxygen vacancy formation, which are known to promote OH^−^ adsorption and O-O bond formation under alkaline conditions [39]. The high-resolution O 1s XPS spectrum of the CMO-120 film (Figure 4c) provides detailed insight into the oxygen chemical environments present at the surface. As shown in the deconvoluted spectrum, the O 1s spectrum can be deconvoluted into three components centered at 529.39 eV (38.71%), 531.24 eV (34.33%), and 532.80 eV (26.96%), which are assigned to lattice oxygen, defect-related oxygen species (associated with oxygen vacancies), and surface-adsorbed oxygen species, respectively. The coexistence of lattice oxygen and a substantial fraction of defect-related oxygen vacancy suggests that the formed electrode surface combines structural stability with chemical reactivity [40]. In particular, oxygen vacancies and hydroxylated oxygen species can enhance OH^−^ adsorption and facilitate surface reconstruction under anodic polarization, which is widely recognized as a key step toward the formation of catalytically active oxyhydroxide-like intermediates during the oxygen evolution reaction [41,42].

### 3.4. Electrochemical Properties of the CoMn_2_O_4_ Electrode Films

**Figure 5:**
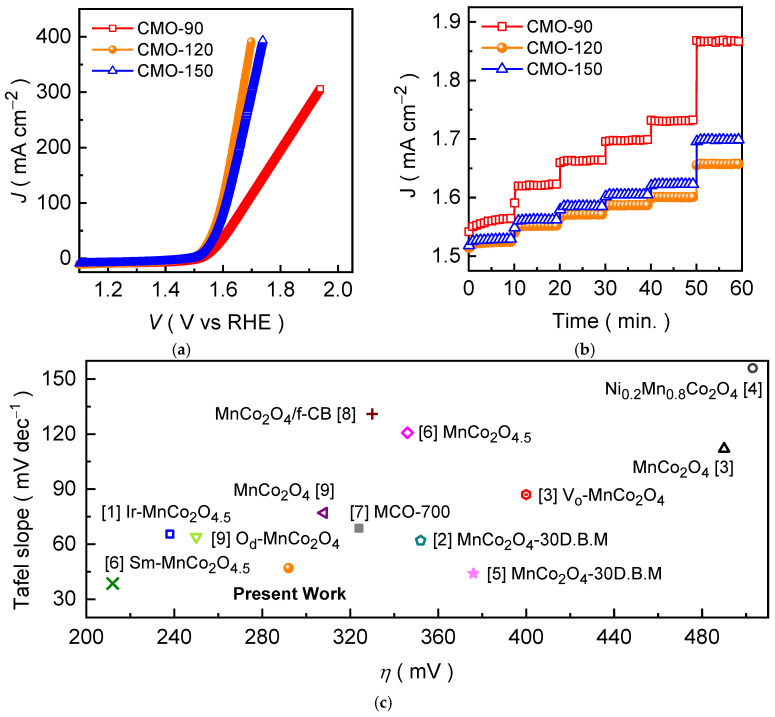
Electrochemical OER performance of CMO-90, CMO-120, and CMO-150 electrode films examined in an alkaline 1.0 M KOH condition. (**a**) LSV curves, (**b**) voltage-step profile, and (**c**) comparative OER performance of CMO-120 electrode and various metal oxide-based catalysts reported at 10 mA cm^−2^ in an alkaline 1.0 M KOH medium.

The electrochemical OER activities of the synthesized CoMn_2_O_4_ electrodes were systematically evaluated in an alkaline 1.0 M KOH medium using the LSV curves benchmarking to comprehensively assess their catalytic efficiencies and practical viability. Figure 5a presents the *iR*-compensated LSV curves of CMO-90, CMO-120, and CMO-150 electrodes recorded at a scan rate of 1 mV s^−1^. All electrodes exhibit typical onset behavior associated with alkaline oxygen evolution, showing negligible current in the pre-OER region followed by a rapid increase in anodic current with increasing potential. A clear distinction in catalytic response emerges among the synthesized electrodes, as the CMO-120 electrode consistently requiring lower overpotentials to achieve the same current density compared with the CMO-90 and CMO-150 electrodes. The CMO-120 electrode attains the current densities of 10, 20, 50, 100, and 300 mA cm^−2^ to achieve the overpotentials of 292, 307, 332, 363, and 434 mV. Whereas the CMO-90 and CMO-150 electrodes require the overpotentials of 321, 344, 387, 454, and 763 mV and 298, 313, 349, 382, and 470 mV, respectively, to drive the same current densities. The systematic downward shift in overpotential from CMO-90 to CMO-120 establishes that moderate hydrothermal heating optimizes the catalyst structure and surface accessibility, while excessive temperature (CMO-150) offers diminished returns due to morphological compaction. The enhanced OER performance of CMO-120 electrode can be attributed to the synergistic interplay between its optimized morphology and surface electronic structure, as revealed by FESEM, XPS, and *ECSA* (Figure S2) analyses. The well-developed, interconnected nanograss architecture provides a large population of electrochemically accessible active sites, as reflected by its increased double-layer capacitance, while maintaining open pathways for electrolyte transport and oxygen release. Concurrently, the coexistence of mixed cobalt (Co^2+^/Co^3+^) and manganese (Mn^2+^/Mn^3+^) oxidation states, together with a significant contribution of defect-related oxygen species, creates a redox-flexible surface that facilitates hydroxide adsorption and stabilizes key OER intermediates. In contrast, the limited surface development of CMO-90 electrode restricts active-site utilization, whereas the excessive growth and partial structural compaction observed for CMO-150 electrode impede mass transport and charge transfer. These combined factors lead to comparatively higher overpotentials and slower reaction kinetics for CMO-90 and CMO-150 electrodes, underscoring the importance of balanced structural and electronic optimization achieved in CMO-120 electrode.

To evaluate the dynamic response and operational stability of the electrodes under progressively increasing current density, a chronopotentiometric step-current test was employed (Figure 5b). Each electrode was subjected to sequential current density increments and the applied current density was constantly maintained for about 10 min to attain a quasi-steady potential. Interestingly, the CMO-120 electrode consistently maintains the lowest potential plateau at each operating current density compared to the CMO-90 and CMO-150 electrodes, reflecting the reduced polarization losses and more efficient charge-transfer kinetics under sustained electrochemical loading. Whereas the CMO-90 electrode exhibits pronounced potential increment with rising current density, indicative of greater kinetic resistance and mass-transport limitations at higher reaction rates. Furthermore, the smooth potential evolution and minimal rise in the overpotential value observed for CMO-120 electrode during current transitions signify a well-balanced electrode–electrolyte interface. The interconnected nanograss morphology of CMO-120 promotes rapid electrolyte permeation, homogeneous current distribution, and efficient removal of oxygen bubbles from the catalyst surface, thereby mitigating concentration polarization and ensuring stable operation even at elevated current densities. For a comparative assessment, the OER activity of the CMO-120 electrode was benchmarked against the Mn-Co spinel and other transition-metal oxides reported for OER in the recent literature (Figure 5c), revealing the reasonably comparative catalytic activity of the CMO-120 electrode with previously reported catalyst electrodes, particularly when both activity and sustained operational behavior are considered.

**Figure 6:**
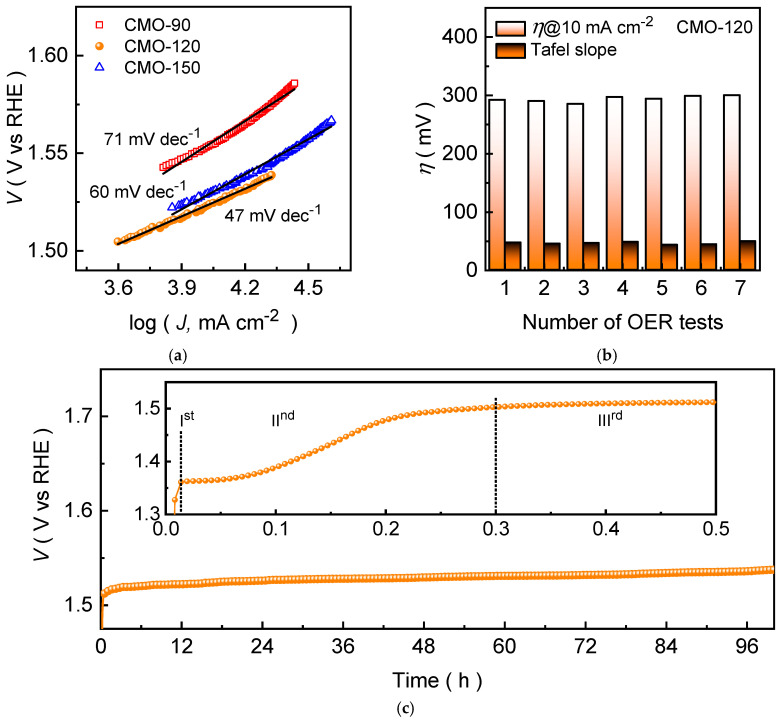
(**a**) Tafel plots, (**b**) OER performance reliability for the CMO-120 electrode, and (**c**) chronopotentiometric stability curve of CMO-120 electrode recorded at a current density of 10 mA cm^−2^ for 100 h.

To further elucidate the intrinsic OER kinetics of the CoMn_2_O_4_ electrode films, Tafel plots were derived from the corresponding polarization curves (Figure 5a). A lower Tafel slope reflects the more favorable electrochemical reaction kinetics during the OER process. Among the all CoMn_2_O_4_ electrodes, the CMO-120 electrode (Figure 6a) exhibits the smallest Tafel slope of 47 mV dec^−1^, confirming the accelerated OER kinetics relative to CMO-190 (71 mV dec^−1^) and CMO-150 (60 mV dec^−1^). This kinetic advantage is a result of the optimized nanograss architecture of CMO-120, which promotes efficient electrolyte permeation and rapid gas release, while simultaneously providing improved charge-transfer pathways through a more continuous and electrochemically accessible network (Figures S2 and S3), and yields the most kinetically favorable CoMn_2_O_4_ electrode for alkaline OER. Thereafter, the reliability of the OER response was evaluated by comparing the OER performance examined under identical testing conditions (Figure 6b and Figure S4). The narrow dispersion in the measured values indicates that the electrochemical behavior is highly reproducible, confirming that the hydrothermal growth process produces consistent electrode films and stable catalyst–substrate electrical contact. Notably, CMO-120 maintains the most consistent performance, with minimal variation across repeated measurements, highlighting the robustness of its nanograss film architecture and its uniform electrochemical utilization. The improved reliability of CMO-120 also aligns with its steady polarization behavior, suggesting that its electrode structure sustains homogeneous current distribution and mitigates localized degradation during operation. The low variation in the measured electrochemical parameters across repeated OER tests demonstrates that all CoMn_2_O_4_ electrodes exhibit the high reproducibility and reliable catalytic behavior, confirming the consistency of the hydrothermal growth process.

The long-term durability of the optimized CMO-120 electrode was assessed by chronopotentiometric testing at a constant current density of 10 mA cm^−2^ for the prolonged duration of 100 h (Figure 6c). The potential-time profile exhibits an initial adjustment stage during the early period of electrolysis (in the inset of Figure 6c). This behavior is attributed to electro-oxidation-driven surface reconstruction, a well-recognized phenomenon in transition-metal-based OER catalysts under alkaline conditions. During this activation process, the surface of CoMn_2_O_4_ undergoes partial transformation into catalytically active Co/Mn (oxy)hydroxide species, accompanied by surface hydroxylation and redistribution of oxidation states. This analysis is in agreement with the post-stability measured XPS spectra (Figure 4), which reveals the increment in the Co^3+^/Mn^3+^ and O_3_ species after prolonged stability in alkaline KOH medium. The post-stability measured EDX spectrum (Figure S5a) further validates the XPS analysis, showcasing the improved concentration of oxygen species, which suggests that extra oxygen is contributed by the electrolyte [29]. Following this initial activation, the operating potential stabilizes and remains nearly constant throughout the 100 h continuous operation, which is consistent with the post-stability measured LSV curve (Figure S5b), demonstrating excellent electrochemical stability. The absence of significant potential alteration indicates that the reconstructed oxyhydroxide layer remains stable during prolonged OER, while the underlying CoMn_2_O_4_ framework preserves structural integrity and efficient electron transport.

## 4. Conclusions

In summary, nanograss-like CoMn_2_O_4_ electrode films were successfully grown directly on stainless-steel substrates via a temperature-controlled hydrothermal approach, and their structure–property–performance relationships for the alkaline OER were systematically investigated. Among the series of electrodes, the CMO-120 emerges as the optimal catalyst, delivering the lowest overpotentials over a wide current density range, favorable OER kinetics with a low Tafel slope of 47 mV dec^−1^, and excellent stability up to 100 h. The superior activity of CMO-120 electrode is primarily attributed to its optimized nanograss architecture, which provides a high electrochemically active surface area while preserving open pathways for electrolyte diffusion and the efficient oxygen bubble release. The EIS and *ECSA* analyses further confirm reduced charge-transfer resistance and an enhanced interfacial charge distribution, leading to accelerated reaction kinetics and minimized polarization losses. Further, the chronopotentiometric testing reveals an initial activation stage, attributed to electro-oxidation-induced surface reconstruction into catalytically active Co/Mn oxyhydroxide species, followed by a highly stable operating potential. This behavior demonstrates that the reconstructed surface remains electrochemically robust, while the underlying CoMn_2_O_4_ framework preserves structural integrity and efficient charge transport. Nonetheless, this work demonstrates that precise temperature control during hydrothermal growth is an effective and scalable strategy for tailoring the morphology, surface chemistry, and electrochemical behavior of Mn-Co spinel oxides, offering practical design guidelines for developing efficient, durable, and binder-free OER electrocatalysts for alkaline water electrolysis.

## Data Availability

The original contributions presented in this study are included in the article/Supplementary Materials. Further inquiries can be directed to the corresponding author.

## Supplementary Materials

The following supporting information can be downloaded at: https://www.mdpi.com/article/10.3390/ma19030542/s1, Figure S1: EDX spectra of (a) CMO-90, (b) CMO-120, and (c) CMO-150 electrode films; Figure S2: Scan rate dependent CV curves of the (a) CMO-90, (b) CMO-120, and (c) CMO-150 electrode films measured in non-Faradaic potential region at different scan rates. (d) “*J* versus *v*” plots obtained at 0.05 V (*vs*. SCE) from non-Faradaic CV curves to calculate the double-layer capacitance and *ECSA*; Figure S3: (a) Nyquist impedance curves and (b) Tank circuit used to fit the EIS curves of the electrode films; Figure S4: Reliability data of (a) CMO-90 and (b) CMO-150 electrodes measured for the series of sample at the same experimental conditions; Figure S5: (a) Post-stability measure EDX spectrum and (b) LSV curves for the CMO-120 electrode film.

## Abbreviations

The following abbreviations are used in this manuscript:

CMO	CoMn_2_O_4_
EIS	Electrochemical impedance spectroscopy
OER	Oxygen evolution reaction
XPS	X-ray photoelectron spectroscopy
LSV	Linear sweep voltammetry
FESEM	Field-emission scanning electron microscopy
XRD	X-ray diffraction
EDX	Energy-dispersive X-ray
CV	Cyclic voltammetry
RHE	Reversible hydrogen electrode
SCE	Saturated calomel electrode
*R*_ct_	Charge transfer resistance
*R*_S_	Solution resistance
*η*	Overpotential
*ECSA*	Electrochemically active surface area
