# Supplementary material for: Morphology and Surface Reconstruction-Driven Catalytic Enhancement in CoMn2O4 for Efficient OER Application
Source: Materials (Basel). 2026 Jan 29;19(3):542. doi: 10.3390/ma19030542 (PMC12897817; doi:10.3390/ma19030542)
Supplement: Supplementary file 1 [file materials-19-00542-s001.zip › materials-4102929-supplementary.docx]

**Supporting Information**

**Morphology and Surface Reconstruction-Driven Catalytic Enhancement in CoMn_2_O_4_ for Efficient OER Application**

Abu Talha Aqueel Ahmed ^1^, Abu Saad Ansari ^2^, Sangeun Cho ^1^**,** and Atanu Jana ^1,*^

^1^ Division of System Semiconductor, Dongguk University, Seoul 04620, Republic of Korea

^2^ Nano Center Indonesia Research Institute, Puspiptek Street, South Tangerang, Banten 15314, Indonesia

**Corresponding Author:** atanujanaic@gmail.com

**Supporting Figures**

(a)

(b)

(c)

**Figure S1.** EDX spectra of (**a**) CMO-90, (**b**) CMO-120, and (**c**) CMO-150 electrode films.

(a)

(b)

(d)

(c)

**Figure S2.** Scan rate dependent CV curves of the (**a**) CMO-90, (**b**) CMO-120, and (**c**) CMO-150 electrode films measured in non-Faradaic potential region at different scan rates. (**d**) “*J* versus *v*” plots obtained at 0.05 V (*vs*. SCE) from non-Faradaic CV curves to calculate the double-layer capacitance and *ECSA*.

The electrochemically active surface area (*ECSA*) of the CoMn_2_O_4_ electrode films (Figure S2) were evaluated by first determining the double-layer capacitance (*C*_dl_) from non-Faradaic CV measurements recorded at multiple scan rates (10-50 mV s^–1^) and then using the relation *ECSA* = *C*_dl_/*C*_e_, where *C*_e_ is the specific capacitance of a smooth surface in alkaline KOH electrolyte. The corresponding “*J* versus $v$” plots were obtained from the Figure S2a-c are provided in Figure S2d. From the comparative slopes, the CMO-120 electrode film exhibits the largest *C*_dl_ of 2.45 mF (and thus the highest *ECSA* of ~ 61 cm^2^), indicating the greatest density of electrochemically accessible active sites among the three electrodes. This trend is consistent with the morphology evolution, as the CMO-90 electrode film shows incomplete developed nanograss coverage (*C*_dl_ = 0.96 mF and *ECSA* = 24 cm^2^, which implies lower accessible area), whereas CMO-150 tends toward overgrowth (*C*_dl_ = 1.97 mF and *ECSA* = ~ 49 cm^2^, which is a result of partially limiting electrolyte access), while CMO-120 achieves an optimal interconnected nanograss network that maximizes exposure of active surface sites. The higher *ECSA* of CMO-120 supports its superior OER performance and faster kinetics relative to CMO-150 and CMO-90 electrode films.

(a)

(b)


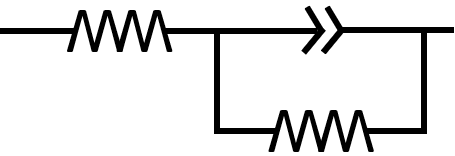


***R_ct_***

***R_S_***

**CPE**

**Figure S3.** (**a**) Nyquist impedance curves and (**b***)* Tank circuit used to fit the EIS curves of the electrode films.

The electrochemical impedance spectroscopy (EIS, Figure S3a) was conducted to evaluate the charge-transfer behavior of the CoMn_2_O_4_ electrode films during OER. The Nyquist plots were fitted using the equivalent circuit shown in Figure S3b, consisting of the solution resistance (*R*_S_) in series with a parallel combination of charge-transfer resistance (*R*_ct_) and a constant phase element (CPE). The CPE accounts for non-ideal capacitive behavior arising from surface roughness and porous nanograss morphology, indicating increased electrochemically active surface area. The *R*_S_ represents the ohmic resistance from the electrolyte and electrode-substrate contact and remains almost comparable for all electrode films, indicating similar testing conditions. The *R*_ct_ reflects the interfacial electron-transfer resistance associated with the OER kinetics. The optimized CMO-120 electrode exhibits the smallest *R*_ct_ of 153 Ω compared to the CMO-150 (171 Ω) and CMO-90 (225 Ω), confirming faster charge transfer and enhanced catalytic activity. Overall, the reduced Rct and enhanced capacitive response of CMO-120 demonstrate improved interfacial charge transport, which is consistent with its lower overpotential, smaller Tafel slope, and superior OER performance.

(a)

(b)

**Figure S4.** Reliability data of (**a**) CMO90 and (**b**) CMO-150 electrode measured for the series of sample at the same experimental conditions.

(a)

(b)

**Figure S5.** (**a**) Post-stability measure EDX spectrum and (**b**) LSV curves for the CMO-120 electrode film.
