# Supplementary material for: Morphology and Surface Reconstruction-Driven Catalytic Enhancement in CoMn2O4 for Efficient OER Application
Source: Materials (Basel). 2026 Jan 29;19(3):542. doi: 10.3390/ma19030542 (PMC12897817; doi:10.3390/ma19030542)
Supplement: Supplementary file 1 [file materials-19-00542-s001.zip › materials-4102929-Supplementary.pdf]

**Supporting Information**

# Morphology and Surface Reconstruction-Driven Catalytic Enhancement in $CoMn_2O_4$ for Efficient OER Application

Abu Talha Aqueel Ahmed [1], Abu Saad Ansari [2], Sangeun Cho [1], and Atanu Jana [1,*]

[1] Division of System Semiconductor, Dongguk University, Seoul 04620, Republic of Korea

[2] Nano Center Indonesia Research Institute, Puspiptek Street, South Tangerang, Banten 15314, Indonesia

**Corresponding Author:** atanujanaic@gmail.com

## Supporting Figures

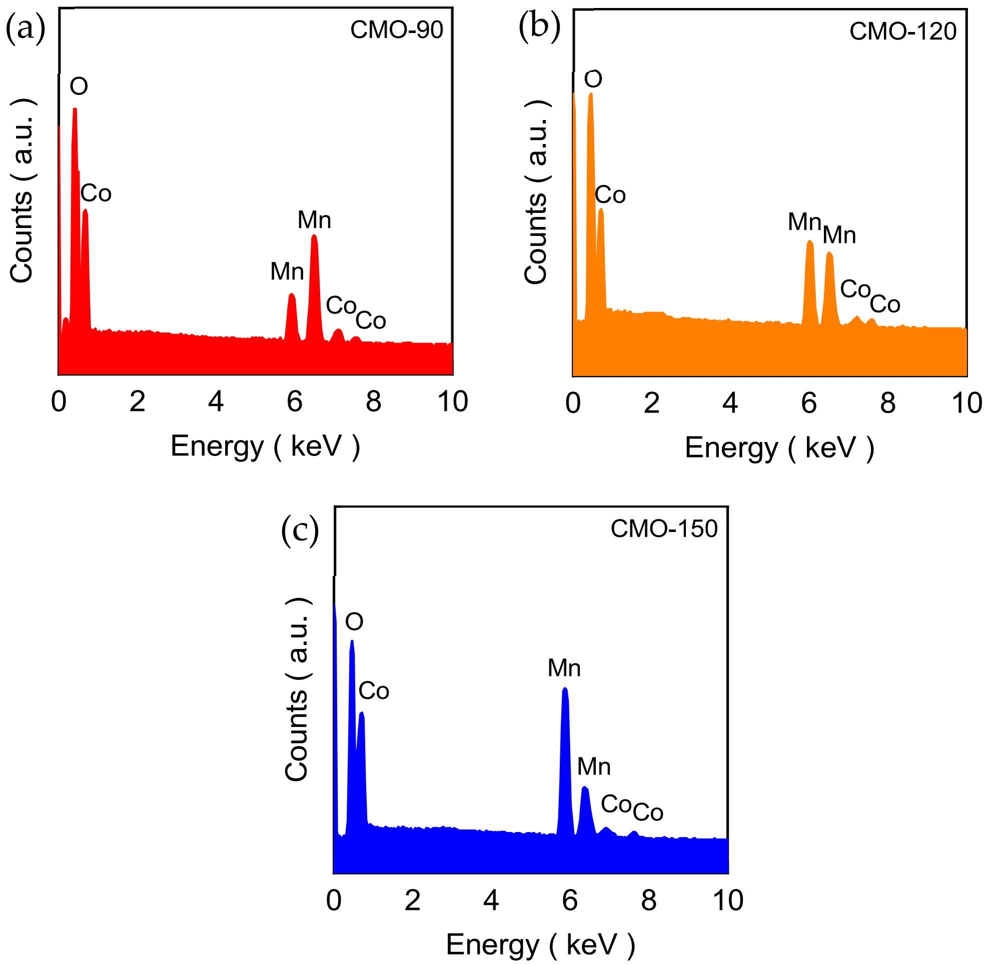


**Figure S1.** EDX spectra of (**a**) CMO-90, (**b**) CMO-120, and (**c**) CMO-150 electrode films.

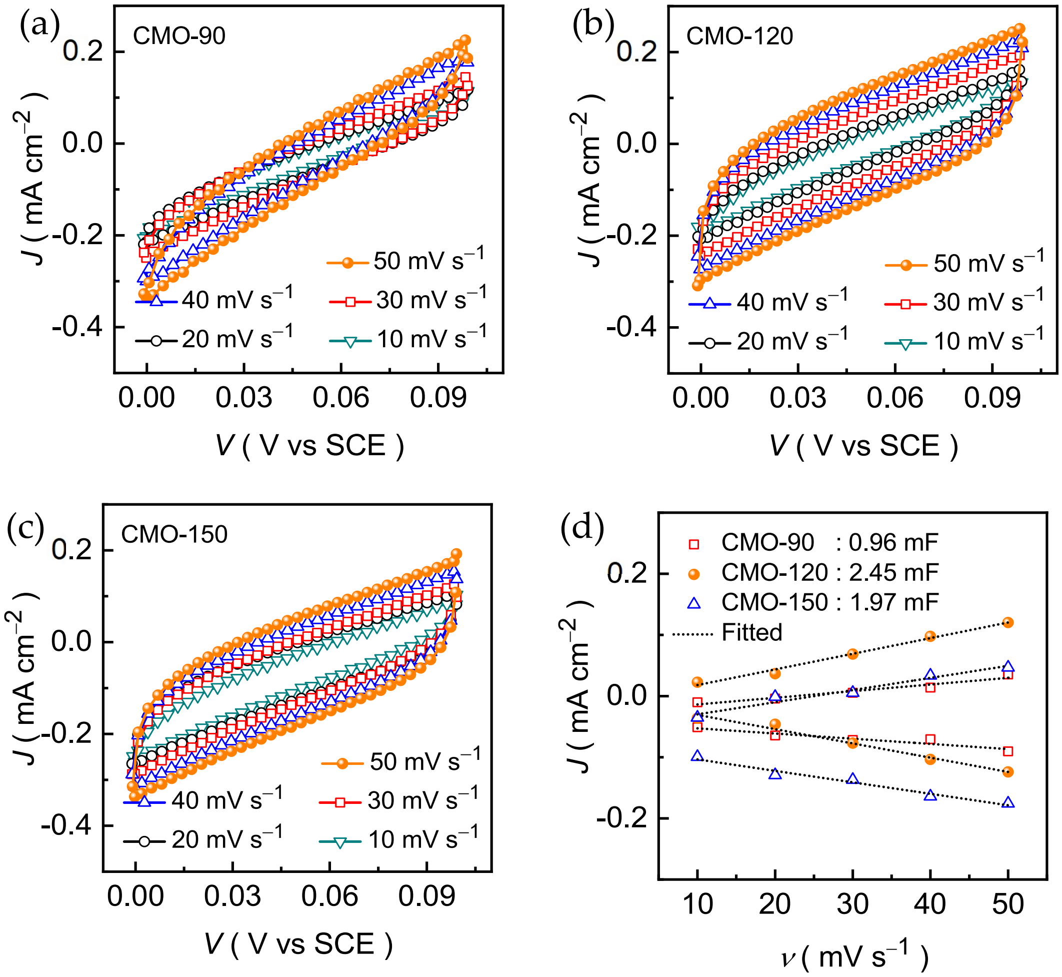


**Figure S2.** Scan rate dependent CV curves of the (**a**) CMO-90, (**b**) CMO-120, and (**c**) CMO-150 electrode films measured in non-Faradaic potential region at different scan rates. (**d**) "*J* versus *v*" plots obtained at 0.05 V (*vs*. SCE) from non-Faradaic CV curves to calculate the double-layer capacitance and *ECSA*.

The electrochemically active surface area (*ECSA*) of the $CoMn_2O_4$ electrode films (Figure S2) were evaluated by first determining the double-layer capacitance ($C_{dl}$) from non-Faradaic CV measurements recorded at multiple scan rates (10-50 mV $s^{-1}$) and then using the relation $ECSA = C_{dl}/C_e$, where $C_e$ is the specific capacitance of a smooth surface in alkaline KOH electrolyte. The corresponding "$J$ versus $v$" plots were obtained from the Figure S2a-c are provided in Figure S2d. From the comparative slopes, the CMO-120 electrode film exhibits the largest $C_{dl}$ of 2.45 mF (and thus the highest *ECSA* of ~ 61 $cm^2$), indicating the greatest density of electrochemically accessible active sites among the three electrodes. This trend is consistent with the morphology evolution, as the CMO-90 electrode film shows incomplete developed nanograss coverage ($C_{dl}$ = 0.96 mF and *ECSA* = 24 $cm^2$, which implies lower accessible area), whereas CMO-150 tends toward overgrowth ($C_{dl}$ = 1.97 mF and *ECSA* = ~ 49 $cm^2$, which is a result of partially limiting electrolyte access), while CMO-120 achieves an optimal interconnected nanograss network that maximizes exposure of active surface sites. The higher *ECSA* of CMO-120 supports its superior OER performance and faster kinetics relative to CMO-150 and CMO-90 electrode films.

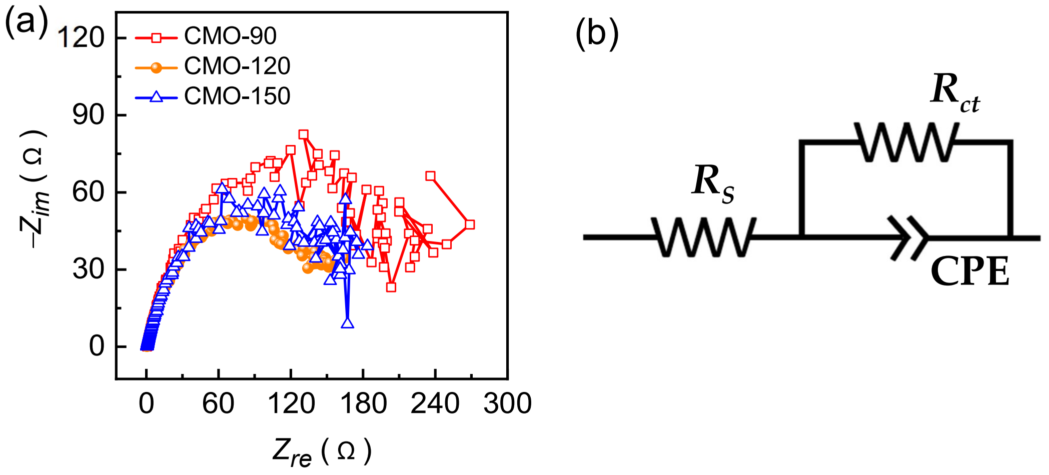


**Figure S3.** (**a**) Nyquist impedance curves and (**b***)* Tank circuit used to fit the EIS curves of the electrode films.

The electrochemical impedance spectroscopy (EIS, Figure S3a) was conducted to evaluate the charge-transfer behavior of the $CoMn_2O_4$ electrode films during OER. The Nyquist plots were fitted using the equivalent circuit shown in Figure S3b, consisting of the solution resistance ($R_S$) in series with a parallel combination of charge-transfer resistance ($R_{ct}$) and a constant phase element (CPE). The CPE accounts for non-ideal capacitive behavior arising from surface roughness and porous nanograss morphology, indicating increased electrochemically active surface area. The $R_S$ represents the ohmic resistance from the electrolyte and electrode-substrate contact and remains almost comparable for all electrode films, indicating similar testing conditions. The $R_{ct}$ reflects the interfacial electron-transfer resistance associated with the OER kinetics. The optimized CMO-120 electrode exhibits the smallest $R_{ct}$ of 153 Ω compared to the CMO-150 (171 Ω) and CMO-90 (225 Ω), confirming faster charge transfer and enhanced catalytic activity. Overall, the reduced Rct and enhanced capacitive response of CMO-120 demonstrate improved interfacial charge transport, which is consistent with its lower overpotential, smaller Tafel slope, and superior OER performance.

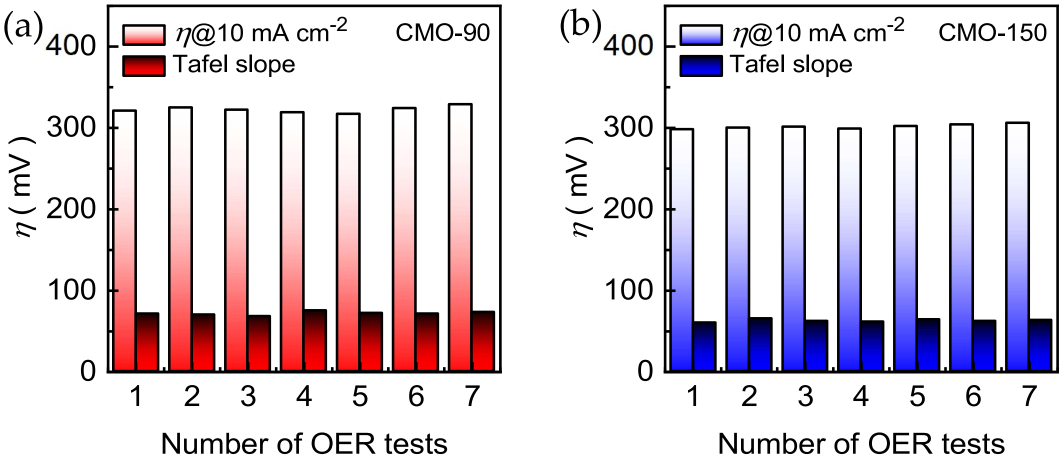


**Figure S4.** Reliability data of (**a**) CMO90 and (**b**) CMO-150 electrode measured for the series of sample at the same experimental conditions.

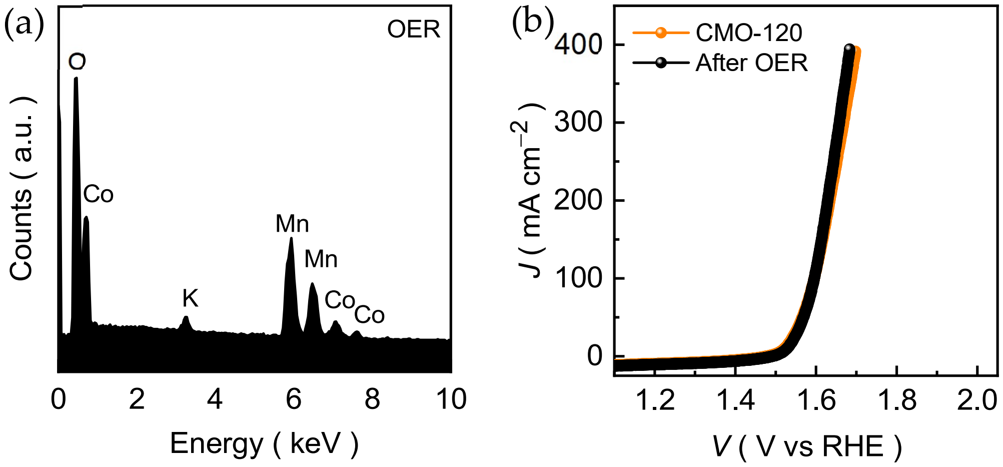


**Figure S5.** (**a**) Post-stability measure EDX spectrum and (**b**) LSV curves for the CMO-120 electrode film.
